# Renal and suprarenal insufficiency secondary to familial Mediterranean fever associated with amyloidosis: a case report

**DOI:** 10.1186/1752-1947-5-390

**Published:** 2011-08-18

**Authors:** Ahmet Burak Toros, Fusun Erdenen, Nagehan Didem Sari, Serkan Gokcay

**Affiliations:** 1 Department of Gastroenterology, Istanbul Education and Research Hospital, Samatya, Fatih, Istanbul, Turkey; 2 Department of Internal Diseases, Istanbul Education and Research Hospital, Istanbul, Turkey; 3 Department of Clinical Microbiology and Infectious Diseases, Istanbul Education and Research Hospital, Istanbul, Turkey

## Abstract

**Introduction:**

Familial Mediterranean fever is an autosomal recessive disease that predominantly affects people of the Mediterranean coast. One of the most frequent complications of the disease is amyloidosis. This clinical entity is known as secondary (also called AA) amyloidosis.

**Case presentation:**

In this report, we describe the case of a 33-year-old Turkish man with familial Mediterranean fever and chronic renal insufficiency. He was admitted to our clinic with symptoms of suprarenal insufficiency. The patient died three months later as a result of cardiac arrest.

**Conclusion:**

Our aim is to make a contribution to the literature by reporting a case of combined insufficiency due to the accumulation of renal and adrenal amyloid in a patient with familial Mediterranean fever, which has very rarely been described in the literature. We hope that adrenal insufficiency, which becomes fatal if not diagnosed and treated rapidly, will come to mind as easily as chronic renal failure in clinical practice.

## Introduction

Familial Mediterranean fever (FMF) is an autoinflammatory inherited autosomal recessive disease observed especially in Jewish, Arabian, Armenian, and Turkish communities [1]. FMF occurs as a result of mutations in the Mediterranean fever (*MEFV*) gene. The *MEFV *gene resides on the 16th chromosome. More than 75 mutations associated with FMF have been found. Pyrin (or marenostrin), the protein expressed by the *MEFV *gene, is basically observed in myelomonocytic cells [2].

The development of renal amyloid is observed during early childhood in the vast majority of patients with FMF and is associated with symptoms such as fever, abdominal pain, and inflammation attacks. Those patients are grouped as phenotype I. However, in some cases, renal amyloid is seen in older children (in the 13- to 15-year-old age group) before the emergence of FMF symptoms. These patients stay asymptomatic until the formation of renal amyloidosis and are classified as phenotype II [3].

Addison's disease is the primary disease of the adrenal glands and is characterized by defects in the secretion of mineralocorticoid and glucocorticoid hormones. The most common cause of Addison's disease is autoimmune or granulomatous destruction of the adrenal glands [4].

FMF is an inherited inflammatory disease characterized by recurrent episodes of fever and polyserositis. AA amyloidosis (secondary amyloidosis), which is specific to FMF, predominantly develops in the kidneys and is characterized by the existence of proteinuria. If left untreated, reactive amyloidosis, the most devastating complication of FMF, may lead to chronic renal failure. Daily treatment with colchicine can prevent both the FMF attacks and amyloid deposition. The effects of FMF on the endocrine system are unclear. However, thyroid and testicular abnormalities secondary to amyloidosis have been reported in patients with FMF [5,6].

AA type amyloid accumulation due to FMF affects the gastrointestinal system and causes malabsorption of food nutrients, which leads to stubborn diarrhea. Since the liver and spleen are often affected, hepatosplenomegaly is observed. Restrictive cardiomyopathy, congestive heart failure, and arrhythmias may occur as a result of cardiac involvement [7]. Addison's disease caused by amyloid infiltration in the adrenal glands and azoospermia and infertility due to the accumulation of amyloid in the testes are observed.

## Case presentation

A 33-year-old Turkish man was admitted to our emergency department with complaints of nausea, vomiting, weakness, diarrhea, and fever up to 38.8°C which had lasted for one month. The patient, who had been diagnosed with FMF at 17 years of age, had been started on a regular hemodialysis program at 29 years of age upon the development of chronic renal failure due to amyloidosis. He was not taking any pills regularly except colchicine tablets.

At his physical examination, his general appearance was apathic and cachectic, his mucous membranes were dry and pale, and his skin was dry and rough with a urochromic color. His blood pressure was 110/70 mmHg, and his heart rate was 81 beats/minute. His heartbeat was rhythmic with auscultation, and there was no additional sound murmur. His abdominal examination showed that his liver extended 5 cm below the costal margin. The hepatic surface was rough and even and was painless on palpation. Traube's space was closed, and the spleen extended 2 cm below the costal margin. His muscles were atrophied at the extremities, and he had shedding hairs and an arteriovenous fistula on the left arm. No other pathological findings were detected during his systemic examinations.

At the time of his first admission to the hospital, his laboratory tests with abnormal values were hemoglobin 9.2 g/dL, hematocrit 26.9%, mean corpuscular volume 79fL, creatinine 8.3 mg/dL, urea 60 mg/dL, albumin 2.1 mg/dL, sodium 132 mmol/L, International Normalized Ratio 1.30, and prothrombin time 15.6 seconds. Hemoculture, stool microscopy, and stool culture were carried out after we observed recurring high fever attacks during his regular hemodialysis sessions, but he did not have high fever at his follow-up examinations. When no pathogenic microorganisms were detected, intravenous ceftriaxone 1 g twice daily was started empirically. Doppler ultrasonography and echocardiography were performed to identify fistulas, but there was still no obvious explanation for his fever.

After a fever that persisted for more than five days, the patient was started on 1 g of intravenous vancomycin once every four days. Abdominal ultrasonography was performed, and hepatosplenomegaly was detected. The liver edge was regular with hepatic parenchyma and showed no pathological finding. His ductus choledochus (common bile duct), intra-hepatic bile ducts, and gallbladder were all normal. Minimal ascites was detected in the abdomen. Examination of the ascites puncture sample produced no findings in favor of tuberculosis. The patient was scheduled for gastroscopy and colonoscopy but had hypoglycemic attacks during the preparation phase. His esophagogastroscopic examination results were within normal ranges. Examination of rectal biopsy samples taken during colonoscopy revealed type AA amyloidosis.

The patient's fever was closely monitored for hypoglycemic episodes and decreased as a result of two weeks of antibiotic therapy. His ocular fundus examination and cranial MRI scans were normal, but he kept on vomiting frequently. During an attack of hypoglycemia, his laboratory results were blood glucose 36 mg/dL, insulin 0.2 μU/mL, C peptide 1.4 ng/mL, sodium 127 mmol/L, and cortisol 7.68 mcg/dL. A Synacthen stimulation test was planned with consultation by an endocrinologist to investigate for adrenal insufficiency. The maximum cortisol value measured was 8.87 μg/dL, and the patient was diagnosed with adrenal insufficiency, so he was started on prednisolone 40 mg per day administered intramuscularly. Subsequently, prednisolone tablets 10 mg/day were started. The patient's diarrhea came to an end, and his hypoglycemia did not recur. The patient's complaints of fatigue and loss of appetite decreased. He was discharged from the hospital with scheduled out-patient clinic monitoring. Three months later he died in the emergency department as a result of cardiac arrest caused by hypokalemia.

## Discussion

FMF is an autoinflammatory autosomal recessive inherited disease and is relatively common in Jewish, Arabian, and Turkish communities. There are usually some difficulties in diagnosing the disease, which generally manifests as fever and abdominal pain attacks due to polyserositis. The disease is often confused with acute appendicitis in patients with abdominal pain. Unnecessary appendectomies performed in these patients represent an important problem. To correctly diagnose the disease, family history, biochemical tests, clinical presentation, and response to colchicine are important. Despite the fact that the Tel Hashomer criteria used for diagnosis do not have the genetic diagnosis title, genetic testing is an important part of the diagnosis today. An *MEFV *gene mutation determination is an auxiliary test for making the diagnosis. Genetic diagnosis is important for patients with type II FMF, especially those with an atypical presentation. However in routine clinical practice, a genetically based diagnosis may not be possible for all patients, since only the 12 most common mutations are checked.

The most significant and frequent complication of FMF is chronic renal deficiency due to the accumulation of renal amyloid. However, the accumulation of amyloid may in fact occur in all kinds of organs and tissues. Although the most common types of amyloid are AA, amyloid light chain amyloidosis, and amyloidosis transthyretin type, particularly AA-type amyloid deposition is seen in patients with FMF. Accumulation of amyloid in the adrenal glands is a rare condition. Also, in clinical practice, it is important to recognize that the most common causes of Addison's disease are granulomatous diseases such as tuberculosis and autoimmunity [8].

Adrenal amyloid accumulation that manifests in the form of Addison's disease leads to nonspecific symptoms. Severe hypotension, sodium loss, hyperkalemia, and hypoglycemia occur. To make the diagnosis, random cortisol level assessments and an adrenocorticotropic hormone stimulation test are used. To rule out the most common factors, antibody level measurement and imaging techniques are used. It is very important to start corticosteroid replacement therapy immediately after Addison's disease is diagnosed.

As for the symptoms of FMF, daily colchicine treatment can reduce the frequency of polyserositis episodes occurring with abdominal pain and prevent amyloid deposition. For patients who are unresponsive to colchicine treatment, interferon α or thalidomide can be applied as alternative forms of treatment [9].

Although amyloid often deposits in the kidneys, it can be seen in any other organ or tissue [10]. Since our patient had FMF with an atypical presentation and was diagnosed late, he was started on colchicine treatment in his early adulthood. Hence, he developed chronic renal failure shortly afterward and had to undergo regular hemodialysis. AA amyloid deposited in his kidneys as well as in his adrenal glands, hence triggering an adrenal crisis, which is quite rare in patients with FMF. When the patient was started on corticosteroid replacement therapy, his diarrhea rapidly disappeared, his fatigue decreased, and his episodes of hypoglycemia ended.

## Conclusion

In the present report, we have tried to make a contribution to the literature by describing the case of a patient with combined insufficiency in FMF due to the accumulation of renal and adrenal amyloid, which has very rarely been reported previously. We hope that adrenal insufficiency, which becomes fatal if not diagnosed and treated rapidly, will come to mind as easily as chronic renal failure in clinical practice.

### Consent

Written informed consent was obtained from the patient's son for publication of this case report and any accompanying images. A copy of the written consent is available for review by the Editor-in-Chief of this journal.

## Competing interests

The authors declare that they have no competing interests.

## Authors' contributions

ABT analyzed and interpreted the patient data regarding the diseases. FE, NDS, and SG were major contributors to the writing of the manuscript. All authors read and approved the final manuscript.
